# Drug Repurposing Prediction and Validation From Clinical Big Data for the Effective Treatment of Interstitial Lung Disease

**DOI:** 10.3389/fphar.2021.635293

**Published:** 2021-09-21

**Authors:** Soni Siswanto, Hiroki Yamamoto, Haruka Furuta, Mone Kobayashi, Takuya Nagashima, Gen Kayanuma, Kazuki Nagayasu, Yumiko Imai, Shuji Kaneko

**Affiliations:** ^1^ Department of Molecular Pharmacology, Graduate School of Pharmaceutical Sciences, Kyoto University, Kyoto, Japan; ^2^ Laboratory of Regulation of Intractable Infectious Diseases, National Institutes of Biomedical Innovation Health and Nutrition, Osaka, Japan

**Keywords:** amiodarone, dabigatran, adverse event, chronic inflammation, pulmonary fibrosis, real-world data, retrospective analysis

## Abstract

Interstitial lung diseases (ILDs) are a group of respiratory disorders characterized by chronic inflammation and fibrosis of the pulmonary interstitial tissues. Although the etiology of ILD remains unclear, some drug treatments are among the primary causes of ILD. In the present study, we analyzed the FDA Adverse Event Reporting System and JMDC Inc. insurance claims to identify a coexisting drug that reduced the incidence of ILD associated with the use of an anti-arrhythmic agent, amiodarone, and found that the thrombin inhibitor dabigatran prevented the amiodarone-induced ILD in both clinical datasets. In an experimental validation of the hypothesis, long-term oral treatment of mice with amiodarone caused a gradual decrease in body weight caused by respiratory insufficiency. In the lungs of amiodarone-treated mice, infiltration of macrophages was observed in parallel with a delayed upregulation of the platelet-derived growth factor receptor α gene. In contrast, co-treatment with dabigatran significantly attenuated these amiodarone-induced changes indicative of ILD. These results suggest that dabigatran is effective in preventing drug-induced ILD. This combinatorial approach of drug repurposing based on clinical big data will pave the way for finding a new treatment with high clinical predictability and a well-defined molecular mechanism.

## Introduction

Interstitial lung diseases (ILDs) are a heterogeneous group of parenchymal lung disorders characterized by varying degrees of inflammation and fibrosis that lead to loss of lung function. Some of these may occur secondary to a known inducer such as adverse drug effects, autoimmune connective tissue disorders, or hypersensitivity to inhaled substances or smoking, whereas idiopathic pulmonary fibrosis (IPF) is a progressive and ultimately fatal, respiratory disorder that has no definitive cause (Barratt et al., 2018; Skeoch et al., 2018). The pathogenic cascade of lung fibrosis is thought to be initiated by perpetual microinjuries to the alveolar epithelium that engenders a dysregulated wound healing response, where immune and inflammatory cells are involved in the fibrotic process with proinflammatory and profibrotic cytokines (Desai et al., 2018). Although much effort has been expended in finding an effective treatment for IPF, only pirfenidone (Schaefer et al., 2011) and nintedanib (Wollin et al., 2015), both having indefinite antifibrotic mechanisms, have been approved for clinical use. The incomplete understanding of the etiology and continued lack of effective treatment makes IPF a disease with considerable unmet need requiring novel approaches to treatment.

Drug repurposing is a systematic approach to find new therapeutic use for already-available drugs (Parvathaneni et al., 2019). For IPF, many repurposing efforts have focused on the fibrotic process with cytokines (Yang et al., 2015; Lu et al., 2017), the involvement of oxidative stress (Sato et al., 2016), or coagulation disturbances accompanied with fibrosis (Howell et al., 2001). In addition, similarities between IPF and cancer with respect to their responses to kinase inhibitors (Staab-Weijnitz and Eickelberg, 2016), and in silico analysis of gene expression signatures (Karatzas et al., 2017) have been utilized to predict candidate compounds. However, the effectiveness proved in animal models of IPF, mostly through bleomycin-induced pulmonary fibrosis (Carrington et al., 2018), has yet to be translated into clinical usefulness.

To improve the clinical predictability of drug repurposing, here we attempted the “reverse translational” approach (Kaneko and Nagashima, 2020) using retrospective analysis of clinical big data, and found a hypothetical drug-drug interaction that ameliorated drug-induced ILD in patients. Previously, we utilized self-reports of adverse events extracted from the FDA Adverse Event Reporting System (FAERS) to investigate the molecular mechanism of quetiapine-induced hyperglycemia (Nagashima et al., 2016). However, the drawback of FAERS is the lack of time stamps and the total number of patients taking the drug of interest. Therefore, we added another source of clinical real-world data, that of insurance claims obtained from JMDC Inc., to enable retrospective analysis for detecting a causal relationship between the use of drugs and the resultant adverse events. The hypothesis was then validated in animal experiments.

## Materials and Methods

### Analysis of the FAERS Database

Adverse event reports from 2004 to 2019 were obtained from the FDA website (https://www.fda.gov/drugs/drug-approvals-and-databases/fda-adverse-event-reporting-system-faers). Duplicated reports were eliminated as previously reported (Banda et al., 2016), and the remaining 11,438,031 reports were analyzed in this study. Arbitrary drug names, including trade names and abbreviations, were manually mapped to unified generic names with Medical Subject Headings (MeSH) descriptor ID. Reports of ILD were defined according to the narrow scope of the standardized MedDRA query “interstitial lung disease” in MedDRA version 23.0. Analysis of the FAERS database was performed as described previously Nagashima et al. (2016). Briefly, adverse event risk was evaluated by calculating the reporting odds ratio (ROR) along with the 95% confidential interval (CI) and *Z* score (see Supplementary Tables S1, S2 in detail). In volcano plots, *Z* scores were used instead of *P*-values to save space.

### Analysis of the JMDC Claims Database

Insurance claims data from January 2005 to August 2019 were purchased from JMDC Inc., (Tokyo, Japan). The dataset contained the medical diagnosis and prescription claims of 7,438,470 anonymized employees and their dependents on a monthly basis. Owing to the features of the general employee population and the national health insurance system in Japan, the patients were mostly aged ≤65 years, and no patients aged ≥75 years were included. Individual diagnoses were assigned according to the International Classification of disease 10 (ICD-10). Cases of ILD were identified by the ICD-10 codes J704, J841, and J849 involving “interstitial pulmonary disease” and “interstitial pneumonia.” According to this definition, there were 20,152 patients with ILD in the JMDC records.

Looking at the time distribution of the first event after enrolment in JMDC, we first defined the run-in period to detect the onset and causality of amiodarone-induced ILD by eliminating patients who had already received a diagnosis of ILD or a prescription with amiodarone before the enrolment of insurance. We also excluded patients who received only amiodarone injection to observe the effects of long-term use of amiodarone. The causal relationship between drug use and ILD was then evaluated using sequence symmetry analysis over an observation period of ±36 months, as described previously by Lai et al. (2017). Cumulative incidences of ILD were compared between the populations with and without concomitant dabigatran using conventional survival analysis (Yokoyama et al., 2018), and the survival curves were represented by Kaplan-Meier curves. Statistical significance was evaluated using a log-rank test, and the Cox proportional regression was used to calculate the hazard ratio. The number at risk shows the number of patients who may have onset of ILD at each month.

### Animals

All animal experiments were approved by the Kyoto University Animal Research Committee in accordance with the ethical guidelines of the Committee. All experiments were designed to minimize the use of animals and the number of experiments. Male C57BL/6J mice (6–7 weeks old, 20–30 g) were purchased from Japan SLC (Shizuoka, Japan). All animals were housed at a constant ambient temperature (22 ± 2°C) on a 12 h light/dark cycle with free access to food and water.

### Reagents and Treatments

Amiodarone hydrochloride was purchased from Nacalai Tesque (Kyoto, Japan), and dabigatran etexilate was purchased from Combi-Blocks (San Diego, CA, United States). Amiodarone and dabigatran were suspended in 0.5% carboxymethylcellulose sodium salt (Nacalai Tesque) just prior to use.

Mice (*n* = 18-28 per group) were randomized to four groups and treated on weekdays orally with amiodarone (300 mg kg^−1^ day^−1^, 5 times per week on weekdays) and dabigatran (60 mg kg^−1^ day^−1^, 5 times per week), or both simultaneously. The vehicle group received the same volume of 0.5% carboxymethylcellulose sodium salt. Body weight was monitored daily, and on day 26, lung tissue was collected for histological screening and gene expression analysis. In a preliminary experiment shown in Supplementary Figure S1, mice were randomized to two groups and treated similarly on weekdays with amiodarone or vehicle, and the lung tissue was collected on days 1, 3, 5, 12, 19, and 26 for gene expression analysis.

### Histological Examination

The left lung tissue was embedded in Tissue-Tek OCT compound (Sakura Finetek, Tokyo, Japan), frozen and stored at −80°C for cryosectioning. The lung was cryosectioned into 10 µm thick sections with a cryostat (Leica CM 1950; Leica Biosystems, Vista, CA, United States) and stored at −80°C. For immunohistochemistry, the sections were immersed in phosphate-buffered saline containing 0.1% Triton X-100 (Nacalai Tesque) for permeabilization and incubated overnight at 4°C with goat polyclonal anti-Iba1 antibody (1:300; 011-27991, Lot CAF1113, Wako, Osaka, Japan), followed by incubation with Alexa Fluor 594-labeled donkey anti-goat IgG (1:200; A11058, Lot 2185074 Invitrogen, Thermo Fisher Scientific, Waltham, MA, United States) for 2 h at room temperature in the dark. After staining nuclei with DAPI Fluoromount-G (SouthernBiotech, Birmingham, AL, United States), images were captured using a confocal fluorescence microscope (Fluoview FV10i; Olympus, Tokyo, Japan). The number of macrophages in a 0.045 mm^2^ field was counted.

### RNA-Seq and Quantitative Reverse Transcriptase Polymerase Chain Reaction

The mRNA expression levels were analyzed using RNA-Seq and qRT-PCR. Total RNA was isolated from the left lung tissue using Isogen reagent (Nippon Gene, Tokyo, Japan).

In RNA-Seq analysis, poly(A)^+^ RNA was selected from total RNA and sequenced using DNBseq (BGI, Shenzhen, China). The clean reads were mapped to the mouse reference genome GRCm38.p6 and the reference gene sequence using HISAT version 2.0.4 (Kim et al., 2015) and Bowtie2 version 2.2.5 (Langmead and Salzberg, 2012), respectively. Gene expression was calculated using RSEM version 1.2.8 (Li and Dewey, 2011) and then log-transformed (for example, expression level of 8 (= 2^3^) becomes 3, expression level of 128 (= 2^7^) becomes 7, and so on). Inflammation-associated, fibrosis-associated, and both-associated gene sets were obtained from Harmonizome (https://maayanlab.cloud/Harmonizome/) (Rouillard et al., 2016). Genes with standardized values, representing a strength of association more than 2.8 between gene and gene set, were used for analysis. Because these gene sets consist of human genes, we transformed them into mouse gene symbols using the biomaRt package (Durinck et al., 2009). Genes with nearly no expression (normalized expression <1) in the vehicle-treated mice were removed from the analysis. The *Z*-score difference of inflammation-associated, fibrosis-associated, and both-associated genes between groups were used for transcriptome profiling. For heatmap visualization, log-transformed gene expression was further standardized (average = 0, variance = 1), and the standardized values of *Z* scores across treatment groups were visualized as a heatmap using Prism 9.2 (GraphPad Software, San Diego, CA, United States). The original sequence datasets were deposited to the NCBI sequence read archive under accession number GSE162229.

For qRT-PCR, isolated RNA was synthetized to cDNA using the ReverTra Ace (Toyobo, Osaka, Japan) and subjected to StepOne Real-Time PCR System (Life Technologies, Carlsbad, CA, United States) with Thunderbird SYBR qPCR Mix (Toyobo). The following conditions were used for the amplification process: 10 min at 95°C, followed by 35 cycles at 95°C for 15 s and 60°C for 1 min. The oligonucleotide primers used for qRT-PCR are listed in Supplementary Table S3. The expression levels of each mRNA were normalized to that of *Pum1* mRNA.

### Statistics

Statistical analyses of the FAERS and JMDC databases were performed with R version 3.4.0 and R studio version 1.3.959 Software (R Foundation for Statistical Computing, Vienna, Austria). The R packages “survival” was used to perform time-series analysis. Log-rank test was used to compare cumulative ILD incidence (Figure 2D). Chi-squared test and Fisher’s exact test were used to compare population characteristics shown in Table 1.

**TABLE 1:** Population characteristics of the patients in the amiodarone cohort of JMDC data.

	Without dabigatran		With dabigatran		*p* Value
Number of patients	1,674	100%	206	100%	
Median age (IQR)	57 (49–62)		57 (52–61)		
Elderly (over 65)	288	17.2%	27	13.1%	0.17
Male	1,365	81.5%	181	87.9%	0.03
Concomitant disease					
Lung cancer	208	12.4%	23	11.2%	0.68
Alcoholism	12	0.7%	2	1.0%	0.66
Renal dysfunction	351	21.0%	41	19.9%	0.79
Diabetes mellitus	1,404	83.9%	182	88.3%	0.12
Concomitant drug					
Bleomycin	0	0.0%	0	0.0%	1.00
Gemcitabine	3	0.2%	0	0.0%	1.00
Tyrosine kinase inhibitors	0	0.0%	0	0.0%	1.00
Anti-EGFR antibodies	1	0.1%	0	0.0%	1.00
mTOR inhibitors	3	0.2%	0	0.0%	1.00
Immune checkpoint inhibitors	3	0.2%	0	0.0%	1.00
Antirheumatic agents	12	0.7%	1	0.5%	1.00
Interferons	2	0.1%	0	0.0%	1.00
Corticosteroids	275	16.4%	20	9.7%	0.02

Statistical analyses of the animal experiments were performed using GraphPad Prism 9.2. These data were presented as the mean ± standard error of mean (SEM). The experimental data of body weight changes (Figure 3A) were analyzed by two-way analysis of variance (ANOVA) with *post hoc* comparison test. Although we did not perform statistical analysis of RNA-Seq data because one sample per treatment group was analyzed, genes whose *Z*-score difference of positive values and negative values were defined as upregulated and downregulated genes, respectively, (Figure 4). The experimental data of quantitative RT-PCR (Figure 5) were analyzed by one-way ANOVA with *post hoc* Tukey’s Multiple Comparison Test.

## Results

### Analysis of FAERS

**FIGURE 1:**
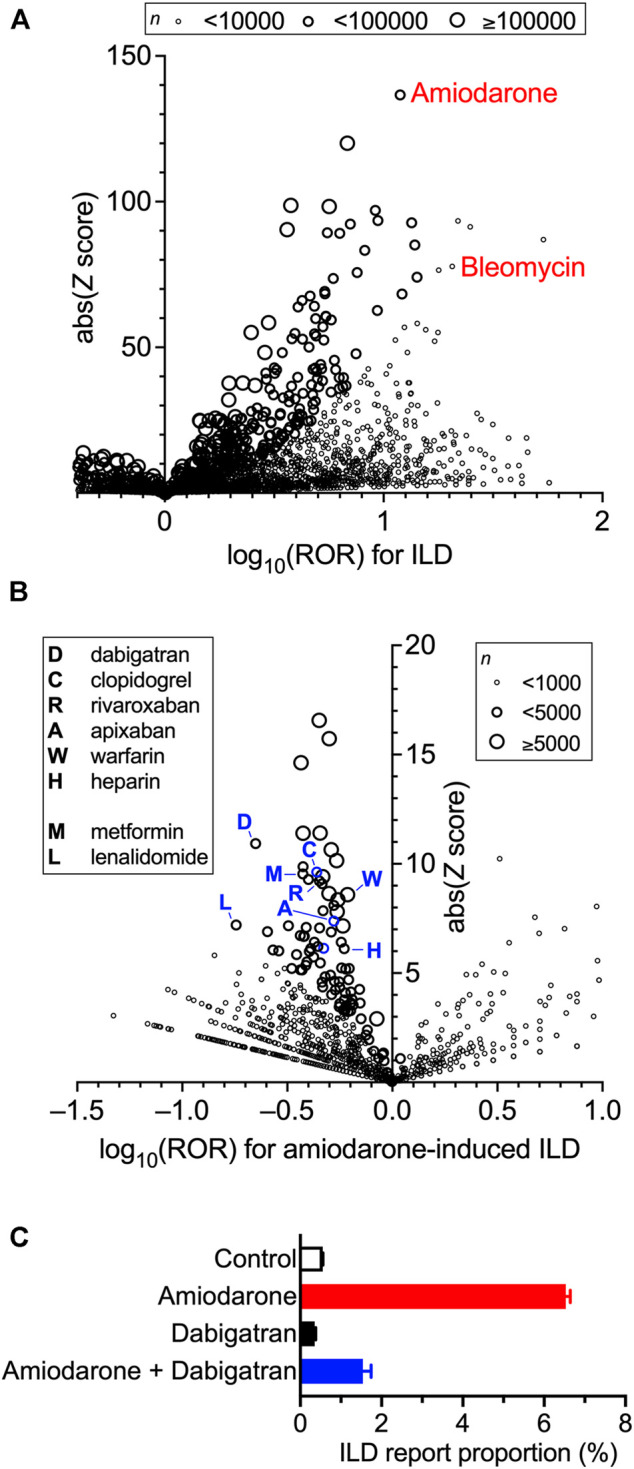
Increased incidence of interstitial lung disease (ILD) with the prescription of drugs and confounding effects of concomitant drugs on amiodarone-induced ILD in the FDA Adverse Event Reporting System (FAERS) data. Volcano plots for visualizing the reporting odds ratio (ROR, on a log scale) and its statistical significance (absolute *Z* score) are shown. Each circle indicates an individual drug, and the size of the circle reflects the number of patients taking the drug. **(A)**, Strong and significant increases in the ROR for ILD were seen in patients using amiodarone and bleomycin. Overall values are presented in Supplementary Table S1. **(B)**, Within the population taking amiodarone, confounding effects of concomitantly used drugs on the incidence of amiodarone-induced ILD were calculated thoroughly and are plotted. Overall values are presented in Supplementary Table S2. **(C)**, Effects of amiodarone, dabigatran, and their combination on the report proportion of ILD in FARES data.

First, we investigated the association between the use of a particular drug and the incidence of ILD in FAERS using disproportionality analysis by calculating each ROR and its *Z* score (Figure 1A). Owing to the known reporting bias and the lack of incidence denominators accompanied by self-reports, these values do not reflect the real incidence rate. Nevertheless, many drugs exhibited a strong association between their use and the emergence of ILD with high RORs and *Z* scores (all data are listed in Supplementary Table S1). Among them, we chose amiodarone because the number of cases was large enough to investigate potential confounders for drug-induced ILD. Bleomycin, an anti-cancer agent frequently used to develop animal models of IPF (Carrington et al., 2018), exhibited higher ROR than amiodarone; however, the number of cases was too small for further analysis.

When the confounding effects of all the drug combinations were calculated and evaluated in a population of amiodarone users, many concomitantly used drugs affected the ROR of amiodarone-induced ILD (Figure 1B). Characteristically, several anticoagulants were found in the moderate-efficacy group, as previously suggested as a possible target for lung inflammation and fibrosis (Chambers, 2008). Among the drugs that lowered ROR, we chose dabigatran for further analysis because of the lowest ROR value (ROR = 0.22) with a sufficient number of cases. Although higher *Z* scores were observed in aspirin, furosemide, and metoprolol, they were omitted because of higher ROR values suggesting lesser effects on ILD (all data are listed in Supplementary Table S2). Metformin, which has been shown to ameliorate bleomycin-induced fibrosis in animals (Sato et al., 2016), exhibited a moderate signal. The most potent lenalidomide was not selected because it is always used with the strong anti-inflammatory agent dexamethasone. Dabigatran did not affect the report proportion of ILD on its own, but in combination with amiodarone, markedly lowered the amiodarone-induced increase in the ROR of ILD (Figure 1C).

### Analysis of JMDC Claims

**FIGURE 2:**
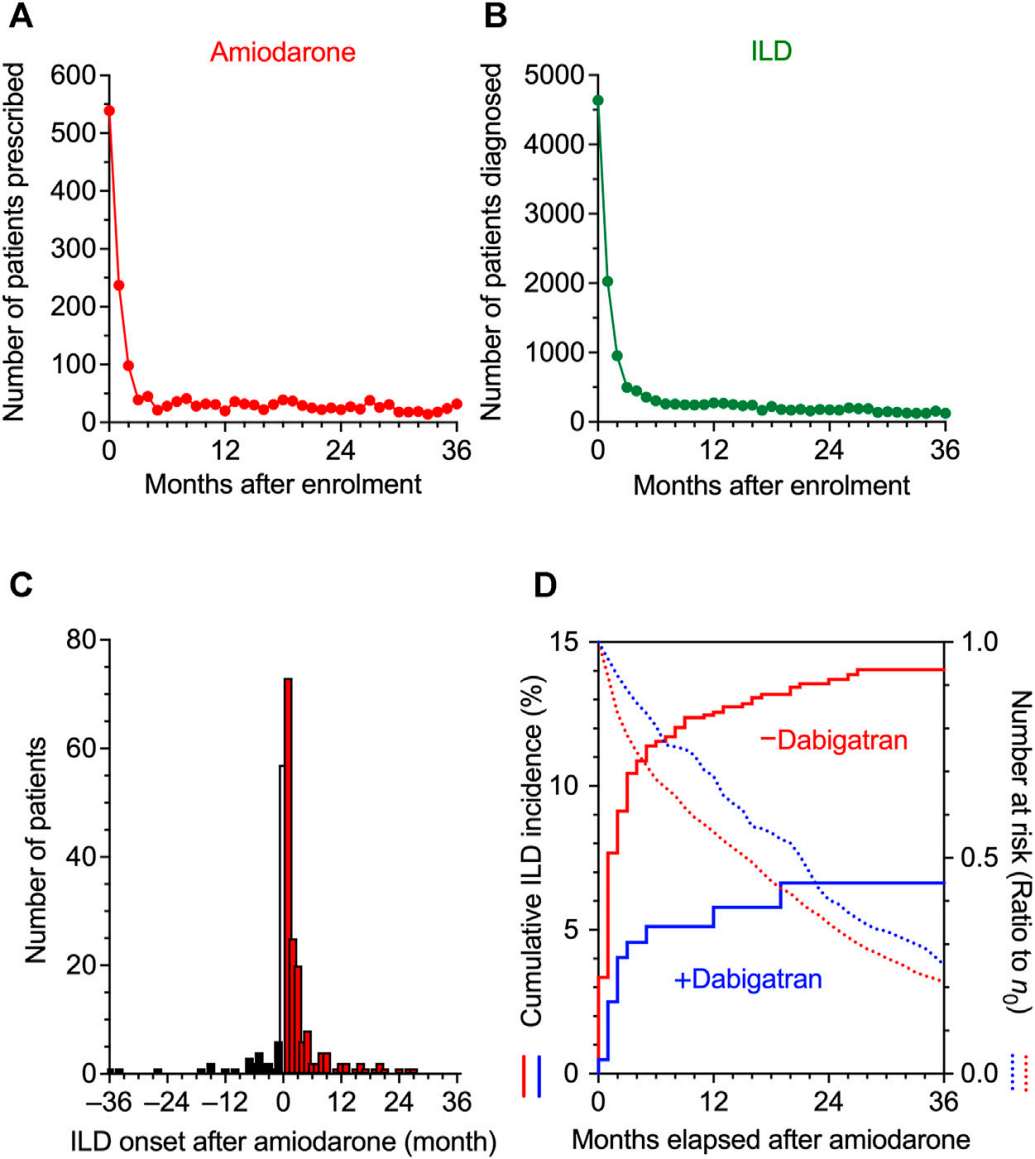
Time distribution of the event in JMDC claims data. **(A,B)**, The intervals from the insurance enrolment of a patient to the first prescription of amiodarone **(A)** and to the initial diagnosis of interstitial lung disease (ILD) **(B)** were analyzed, and the number of patients is shown on a monthly basis. From these data, patients who had been enrolled in health insurance for 0–2 months were omitted in the following analysis to allow a run-in period. **(C)**, Sequence symmetry analysis showing the causal relationship between the start of amiodarone treatment and the onset of ILD in an observation period of ±36 months. At month 0, the precise chronological order was unknown, and the data were omitted from the analysis. **(D)**, Kaplan-Meier curves for the cumulative incidence ratio of ILD in patients taking amiodarone are shown individually in two populations, one without (red) and one with (blue) co-prescribed dabigatran. Dotted lines show the number of patients at risk as a ratio to the initial number (*n*_0_) of patients.

To investigate the consequences of the use of amiodarone and the onset of ILD in clinical records, we analyzed JMDC insurance claims data. Looking at the time distribution of the first event after enrolment of patients in JMDC, the number of patients initially prescribed amiodarone or diagnosed with ILD was much higher during the first 2 months than in subsequent months and became stable after 3 months (Figures 2A,B). These results suggest that there may be a considerable number of patients who received a prescription of amiodarone or diagnosis of ILD before or just after enrolment, and that these patients should be eliminated from the cohort study to evaluate the timeline of amiodarone-induced ILD. Therefore, after removing patients who received a prescription with amiodarone or diagnosis of ILD during the 0 to 2 months run-in period, patients who received amiodarone thereafter were regarded as a cohort. In the amiodarone cohort (*n* = 1,880), a causal association between the use of amiodarone and the onset of ILD was detected with an adjusted sequence ratio of 7.32 (95% CI: 5.13–11.8) in a sequence symmetry analysis (Figure 2C).

We then divided the amiodarone cohort into two populations that received dabigatran (*n* = 206) or not (*n* = 1,674). The population characteristics with regard to age, gender, concomitant diseases, or drugs that are risk factors for ILD (Yamada et al., 2007; Skeoch et al., 2018) were similar (Table 1). The average daily dose of amiodarone was comparably low in the dabigatran-prescribed group (Table 2). However, we speculate that the plasma concentration of amiodarone was maintained within a limited range by mandatory therapeutic drug monitoring in both populations, as the plasma concentration of amiodarone is known to be increased in the presence of dabigatran etexilate by a competitive interaction with P-glycoprotein (Stöllberger and Finsterer, 2015). On the contrary, the cumulative dose and administration period of amiodarone were higher and longer, respectively, in the patients prescribed dabigatran, reflecting a lower number of dropouts as described later. The administration period of dabigatran was longer than that of amiodarone.

**TABLE 2:** Daily and cumulative doses during the administration periods of amiodarone and dabigatran in the amiodarone cohort of JMDC data Median value, inter-quarter range (IQR) and minimum-maximum ranges are shown for each population.

	Without dabigatran		With dabigatran			
	Amiodarone		Amiodarone		Dabigatran	
	Median (IQR)	Range	Median (IQR)	Range	Median (IQR)	Range
Average daily dose (mg)	165 (102–201)	5–788	127 (100–200)	51–454	264 (220–300)	75–300
Cumulative dose (mg)	16,900 (6,200–47,200)	100–667,200	20,300 (8,800–57,975)	1,100–367,350	71,165 (14,835–180,150)	110–823,500
Administration period (day)	116 (35–388)	1–3,737	166 (61–471)	7–2,816	267 (60–772)	1–2,996

Comparing these two populations, Kaplan-Meier analysis and Cox proportional hazards modeling indicated that the combination of dabigatran decreased the amiodarone-induced incidence of ILD with a significant hazard ratio of 0.43 (95% CI: 0.24–0.77, *p* = 0.004 in the log-rank test, Figure 2D). In parallel, the decay in the number of remaining patients at risk was also slowed by dabigatran in the amiodarone cohort, suggesting that dropout from long-term amiodarone treatment was comparably avoided by the use of dabigatran. As a result, the 3 years incidence proportion of ILD decreased from 12.4 to 5.8% with dabigatran (Table 3). In 30 patients without dabigatran, a more severe phenotype accompanied with fibrosis was observed. These results in real-world patient data demonstrate that concomitant use of dabigatran during the treatment of arrhythmia with amiodarone, as an unintended consequence, prevented the onset of drug-induced ILD, leading to pulmonary fibrosis.

**TABLE 3:** Diagnosis of interstitial lung disease (ILD) given to patients taking amiodarone in JMDC data.

	Without dabigatran	With dabigatran
Number of ILD cases in 3 years (%)	208 (12.4%)	12 (5.8%)
Interstitial pulmonary disease, unspecified (J849)	167 (10.0%)	12 (5.8%)
Other interstitial pulmonary diseases with fibrosis (J841)	30 (1.7%)	0
Drug-induced interstitial lung disorders, unspecified (J704)	13 (0.8%)	0

### Effects of Dabigatran on Amiodarone-Induced ILD Model in Mice

**FIGURE 3:**
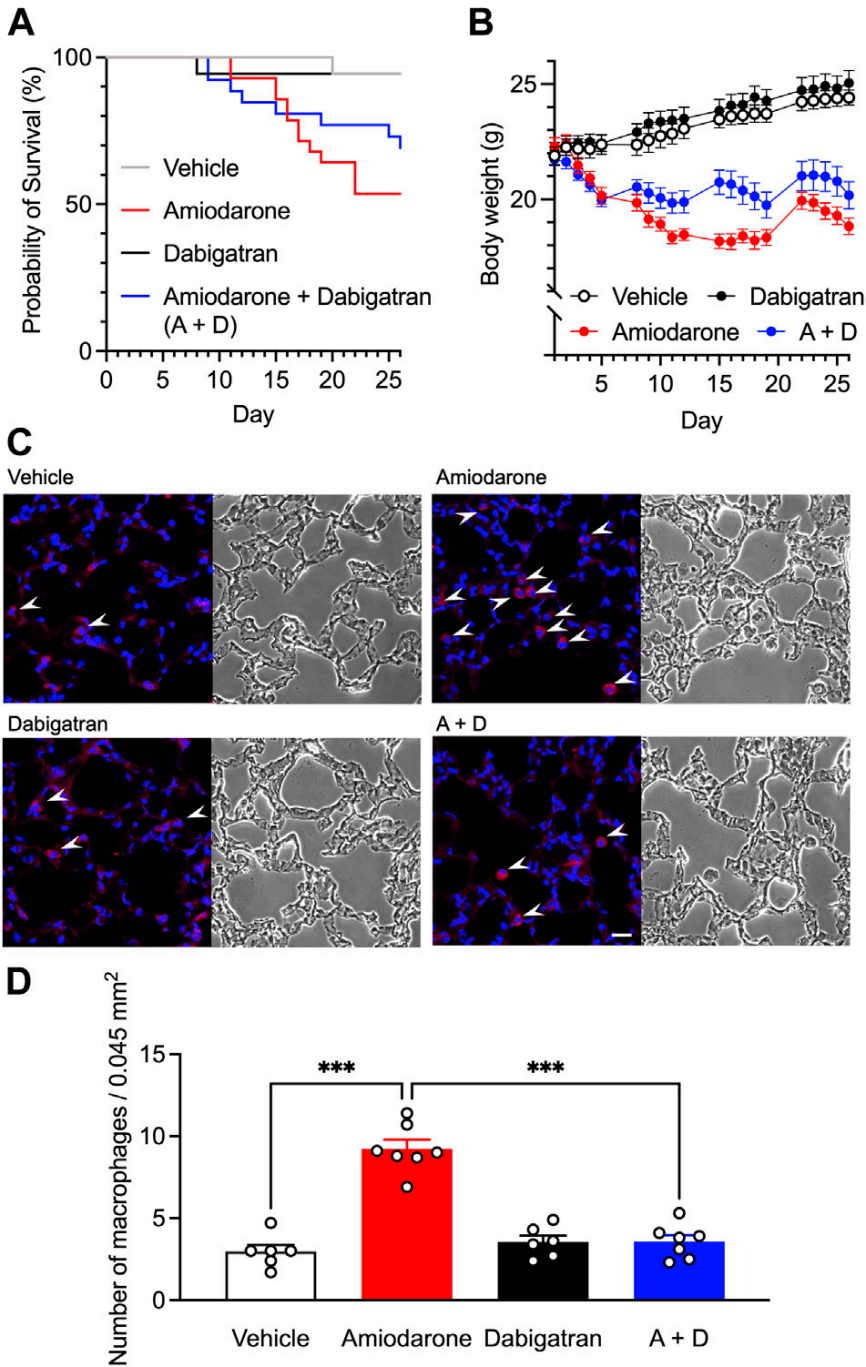
Effects of dabigatran on amiodarone-induced lung toxicity. Kaplan-Meier survival data **(A)** and the body weight changes **(B)** of the male C57BL/6J mice orally treated with amiodarone (300 mg kg^−1^ day^−1^) and dabigatran (60 mg kg^−1^ day^−1^) or both (A + D) 5 times per week on weekdays for 4 weeks (*n* = 18–28 per group). Body weight data are shown as the mean ± SEM of those survived 26 days (*n* = 15–18 per group). Statistical significance was tested by two-way ANOVA with multiple comparisons. **(C)**, Representative images of Iba1-immunostaining (left) and bright field (right) of mouse lung section on day 26 after chronic oral treatment with amiodarone and dabigatran or both. White arrowheads indicate alveolar macrophage infiltration. Scale bar = 20 μm. **(D)**, Numbers of Iba1-positive macrophages in the immunostained images (*n* = 6–7 per group). Statistical significance was tested by two-way ANOVA with Tukey’s multiple comparisons.

Previous studies have shown that inhibition of thrombin action exerts protective effects against bleomycin-induced fibrosis in rats (Howell et al., 2001), and that dabigatran etexilate is orally effective in preventing inflammatory and fibrotic changes by bleomycin in mice (Bogatkevich et al., 2011). To confirm whether dabigatran also mitigates amiodarone-induced ILD in animals, we chronically treated mice with amiodarone and monitored the body weight by which drug-induced respiratory insufficiency with the use of amiodarone or bleomycin can be noninvasively detected (Costa-Jussa et al., 1984; Cowley et al., 2019). A scheduled administration of amiodarone (300 mg kg^−1^ day^−1^ p.o., 5 times per week on weekdays for 4 weeks) caused a decrease in body weight of mice after several days that caused gradual death, probably reflecting lung toxicity (Figures 3A,B). Dabigatran (60 mg kg^−1^ day^−1^ p.o., same schedule) treatment alone did not affect a natural increase in the body weight of mice; however, when co-administered with amiodarone, the amiodarone-induced decrease in body weight was significantly inhibited after the second week (two-way ANOVA, day: *F*_2.7,167_ = 14.4, *p* < 0.001, treatment: *F*_3,61_ = 28.6, *p* < 0.001, interaction: *F*_57,1159_ = 16.7, *p* < 0.001) with a decrease in death rate. Histologically, chronic amiodarone treatment caused an increase in the number of Iba1-positive macrophages in the alveolar space (Figure 3C). However, no apparent accumulation of the extracellular matrix was observed in bright field images and Sirius red staining of lung tissue taken from amiodarone-treated mice (Figure 3C and Supplementary Figure S1A). Dabigatran treatment did not affect the histology by itself; however, with cotreatment, a significant decrease in the number of macrophages was observed (one-way ANOVA, *F*_3,22_ = 45.3, *p* < 0.001, Figure 3D).

**FIGURE 4:**
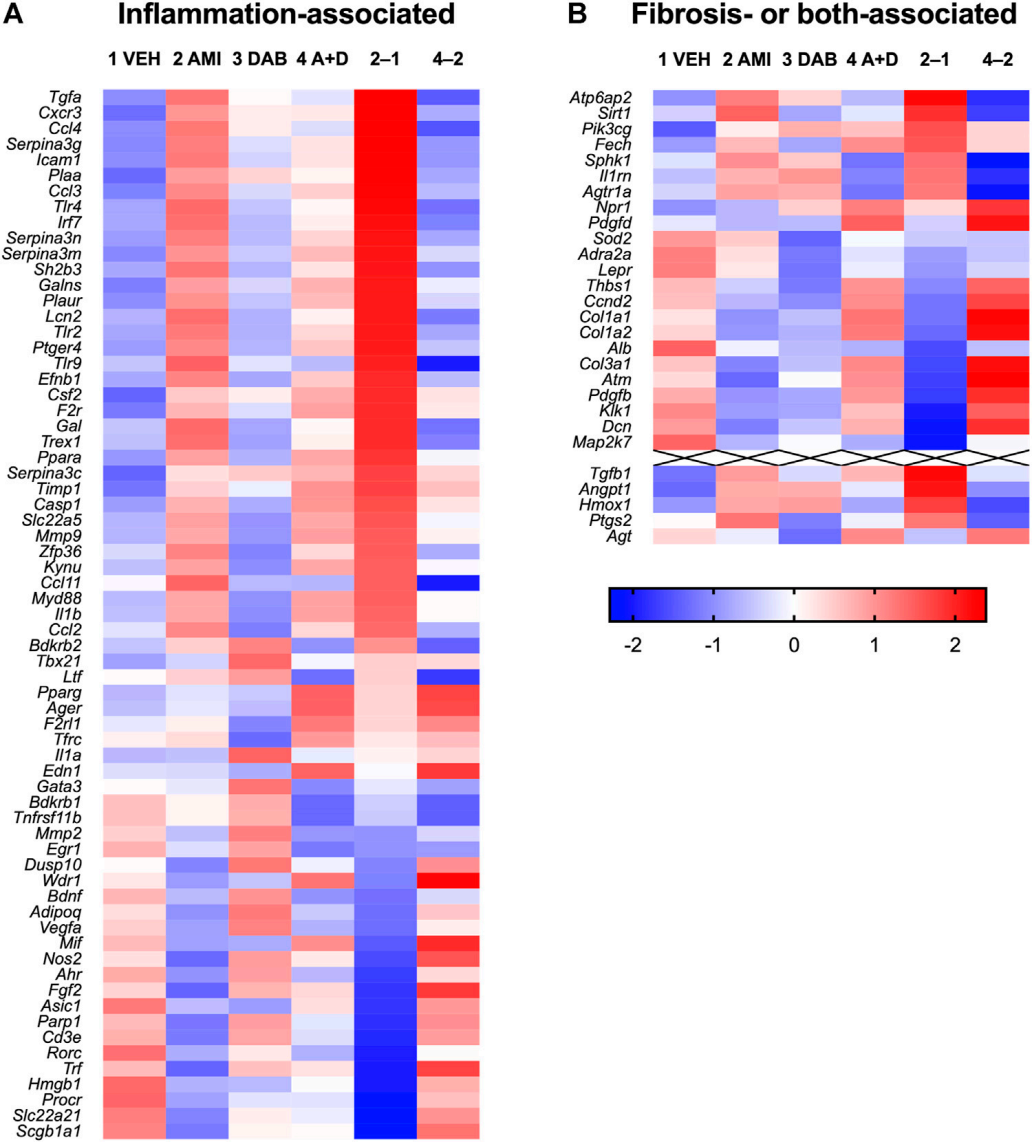
Heatmap of gene expression in the lung tissue (on day 26) of mice treated with vehicle (1 VEH), amiodarone (2 AMI), dabigatran (3 DAB), or amiodarone + dabigatran (4A + D). Log2-expression values calculated from RNA-Seq results are standardized across treatment groups. Red and blue colors represent larger and smaller, respectively, standardized values in *Z* scores. In addition, the differences between AMI and VEH groups (2–1) and A + D and AMI groups (4–2) are shown. Genes are ordered by the difference between the AMI and VEH groups (2–1) from high to low separately in inflammation- **(A)** and fibrosis- or both- **(B)** associated gene sets.

To clarify the molecular bases underlying respiratory insufficiency accompanied with chronic amiodarone, we analyzed gene expression changes in the lung tissue using RNA-Seq, especially focusing on gene families related to inflammation and fibrosis. Among the genes associated with inflammation in Harmonizome (Rouillard et al., 2016), 67 genes were detected in the vehicle-treated mouse lung tissue on day 26 (Figure 4A), and 43 genes were upregulated in the amiodarone-treated group. Dabigatran did not cause any marked changes by itself but reversed the upregulation in 29 genes (67%). These include functional genes encoding proinflammatory cytokines/chemokines (*Tgfa*, *Cxcr3*, and *Ccl2/3/4/11*), inflammatory mediators (phospholipase A_2_ activating protein *Plaa* and prostanoid EP_4_ receptor *Ptger4*), innate immunity-related proteins (Toll-like receptors *Tlr2/4/9* and lipocalin *Lcn2*), cell adhesion molecules (*Icam1* and *Efnb1*), extracellular matrix degrading enzymes (*Mmp2/9* and *Galns*) and their inhibitors (*Timp1*, *Serpina3g/m/n*). In contrast, there were few changes in the expression of fibrosis-associated genes, except in some cases, such as transforming growth factor-β1 *Tgfb1*, V-ATPase *Atp6ap2*, and sirtuin *Sirt1* (Figure 4B). These results suggest that our amiodarone model was in a chronic inflammation stage prior to fibrotic changes in the lung structure, and that dabigatran specifically prevented the proinflammatory changes that deteriorated respiratory function. No apparent increase in the expression of profibrotic genes in chronological qRT-PCR supported this notion (Supplementary Figure S1B).

### Molecular Mechanisms of Dabigatran on Amiodarone-Induced ILD

It has been shown that proteinase-activated receptor 1 (PAR1) is involved both in the bleomycin-induced pulmonary fibrosis (Howell et al., 2005) and in the antifibrotic action of dabigatran (Bogatkevich et al., 2009). As increased risk of bleeding is an inevitable consequence of the use of dabigatran or direct inhibitor of PAR1 (Ramachandran and Hollenberg, 2008), it is worthwhile seeking other cellular signals that are uncoupled from the hemostatic pathway to find the potential therapeutic target of chronic inflammation in the mouse amiodarone model.

**FIGURE 5:**
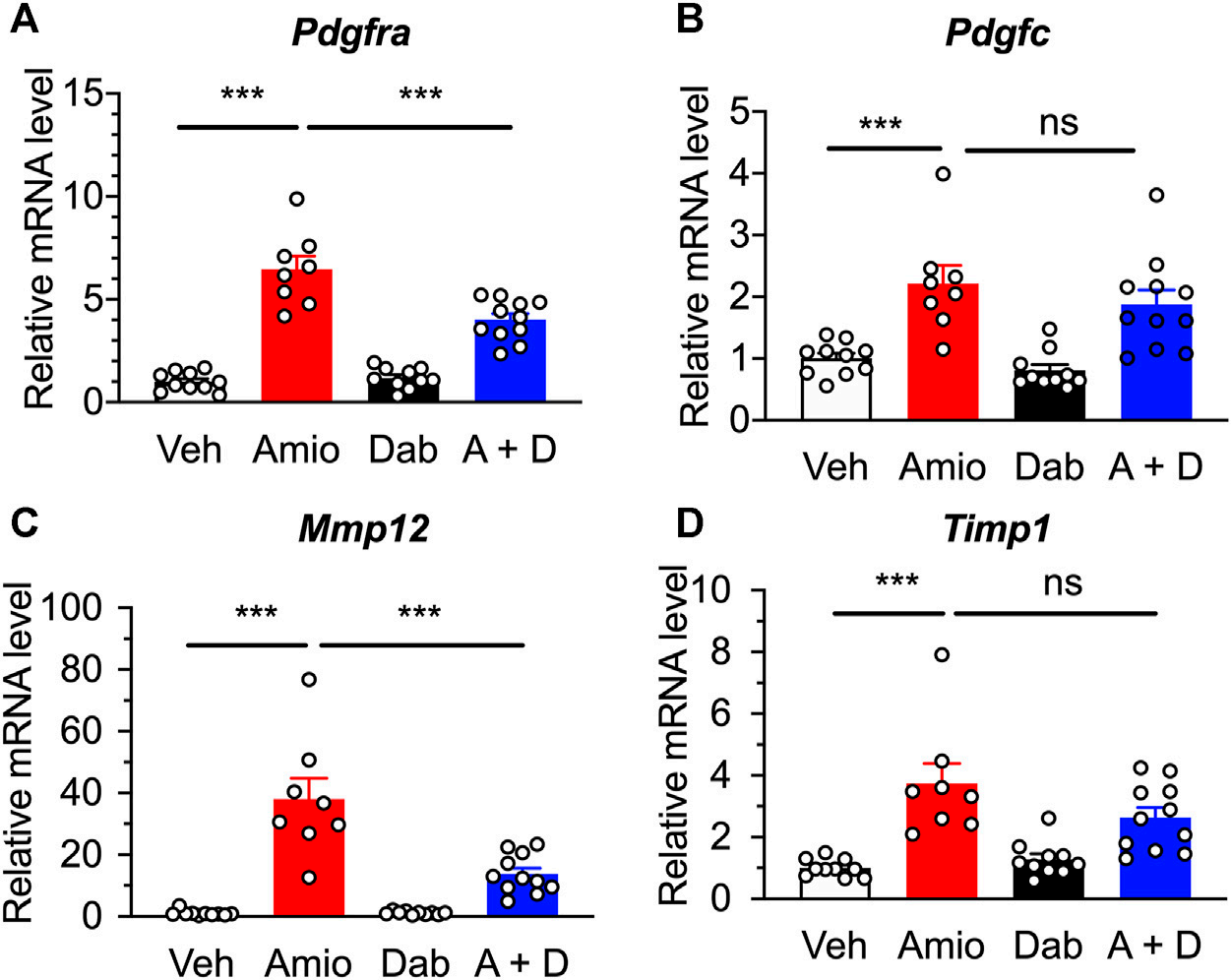
Quantitative RT-PCR results showing the expression of inflammation-associated genes in the lung of mice after a repetitive, oral treatment with amiodarone (Amio, 300 mg kg^−1^ day^−1^) and dabigatran (Dab, 60 mg kg^−1^ day^−1^) or both (A + D) 5 times per week for 4 weeks. Expression levels of *Pdgfra* **(A)**, *Pdgfc* **(B)**, *Mmp12* **(C)**, and *Timp1* **(D)** were normalized to the expression of *Pum1* in each sample from individual mouse (*n* = 8–11), and are represented as the relative ratio to those of vehicle group (Veh). Individual data are shown with the mean ± SEM. Statistical significance was tested by one-way ANOVA with Tukey’s multiple comparisons. ***, *p* < 0.001; ns, not significant.

Based on RNA-Seq data, we searched for promising targets using qRT-PCR, and found that the expression of platelet-derived growth factor receptor α (PDGFRα) mRNA *Pdgfra* was greatly increased in the lung tissue after the 4 weeks treatment of mice with amiodarone compared with vehicle control, and that the upregulation was significantly inhibited by co-treatment with dabigatran, although dabigatran by itself unchanged the expression (Figure 5A). In parallel, the gene *Pdgfc* encoding one of the ligands of PDGFRα was also upregulated by amiodarone; however, the increase was not affected by co-treatment with dabigatran (Figure 5B), suggesting that the action sites of dabigatran are located downstream of those of amiodarone evoking the PDGF signal pathway.

In addition to the increase in cytokines and growth factors, pulmonary fibrosis is associated with increased production of matrix metalloproteinases (MMPs) and tissue inhibitors of matrix metalloproteinases (TIMPs), which are important regulators of connective tissue homeostasis (Malaviya et al., 2020). In the lung tissue of our model, mRNAs for *Mmp12* and *Timp1* were upregulated by amiodarone to 38-fold and 4-fold, respectively, higher than those of vehicle-treated mice (Figures 5C,D). Co-treatment with dabigatran significantly inhibited *Mmp12,* but not *Timp1*, whereas dabigatran by itself did not have any effect on the expression of them.

## Discussion

In the present study, we show for the first time that the direct thrombin inhibitor dabigatran mitigates ILD induced by the long-term use of amiodarone in humans. The finding was observed in two independent clinical big data, and the hypothesis was validated using an animal study with respect to weight loss probably accompanied by respiratory insufficiency as well as changes in pulmonary histology and gene expression.

Data mining using FAERS has unveiled several unexpected drug-drug interactions as a beneficial clinical effect on adverse events (Zhao et al., 2013; Nagashima et al., 2016). In those analyses, the association between drugs and adverse events was strong enough owing to the large size of the data set, regardless of the diversity of clinical situations. In this study, only amiodarone caused a large ROR signal for ILD in more than tens of thousands of cases, which enabled further data mining of various confounding factors with high sensitivity. However, a much smaller number of reports for bleomycin made it difficult to validate several hypotheses of drug repurposing proposed by the use of bleomycin as an IPF model. To date, many drugs have been proposed to ameliorate bleomycin-induced pulmonary fibrosis in animals. Among these targets, blood coagulation factor receptors are key candidates, as these receptors play central roles in influencing inflammatory and fibrotic responses (Lin et al., 2017), although warfarin was ineffective in a clinical trial (Noth et al., 2012). Consistent with the previous findings, several anticoagulants were found to lower the ROR of ILD in the present FAERS analysis. Among them, dabigatran exhibited the lowest ROR signal.

The hypothesis derived from FAERS was further validated in a retrospective cohort analysis using JMDC claims data. In contrast to the electronic health records of patients, JMDC insurance claims data are on a monthly basis, and the exact dates of prescription or diagnosis are unavailable as part of the deidentification process of personal data. Therefore, such data cannot be used to analyze the onset of acute adverse events. However, a considerable proportion of amiodarone-induced ILD arises slowly a month after prescription, and we can detect the onset of ILD. In Japan, low-dose amiodarone therapy with a daily dose of less than 200 mg has been recommended since 1987, and reflecting this situation, the 3 years incidence of ILD was reported to be 7.8% in a university hospital with an average daily dose of 141 mg (Yamada et al., 2007). In the present study, both cumulative incidence of ILD and the average dose were statistically comparable with those in the previous study, warranting the use of JMDC data for retrospective cohort analysis of amiodarone-induced ILD.

Among the patients taking amiodarone, approximately 10% of patients used dabigatran with a longer average period than that of amiodarone, and about half of the patients evaded ILD within 3 years. Accordingly, the number of patients taking corticosteroids was significantly lower in the with-dabigatran group than in the without-dabigatran group (Table 1). In addition, the symptoms of ILD seemed milder in the dabigatran group, as evident from the lack of diagnosis with J841 (ICD-10), including fibrosis. Because the population characteristics of patients with or without dabigatran were almost equivalent, these results strongly suggest that the direct thrombin inhibitor is capable of inhibiting the initiation of lung inflammation and fibrosis by amiodarone in humans. The usefulness of dabigatran is not limited to the prevention of drug-induced ILD, as ILD is a frequent complication of fibrotic disease such as systemic sclerosis (SSc). A recent, prospective, open-label clinical study indicated that dabigatran appears to be safe and well tolerated in patients with SSc-associated ILD (Silver et al., 2019).

Using drug-induced disease models of animals, much effort has been directed toward finding an effective drug for ILD or IPF from among the drugs approved for clinical use. Dabigatran has been shown to be effective in bleomycin-induced fibrosis by mediating the PAR1–α_v_β_6_ integrin–TGFβ1 pathway that is activated by thrombin derived from vascular leak (Shea et al., 2017). In our study, however, a 4 weeks oral treatment of mice with amiodarone caused chronic inflammation but not fibrotic changes at the interstitial space of the lung. Clinical data from JMDC also supported that human ILD induced by amiodarone mainly comprises interstitial alveolitis but not fibrosis. Therefore, we hypothesized that inflammation precedes fibrotic changes by chronic amiodarone and searched for other critical biomolecules involved in the amiodarone-induced ILD. Using the mouse model, we verified that ILD caused by amiodarone can be prevented by co-treatment with dabigatran. Moreover, we found that the expression of PDGFRα was oppositely regulated by amiodarone and dabigatran, whereas the upstream PDGF-C gene was only affected by amiodarone. These results imply that the PDGFRα gene is the key molecule that dabigatran competes with for the proinflammatory and profibrotic processes activated by amiodarone.

PDGFRα is a critical growth factor receptor that stimulates the proliferation and migration of fibroblasts and myofibroblasts, leading to the production of extracellular matrix (Luzina et al., 2015). It has been reported that thrombin upregulates PDGFRα mRNA and protein expression in human lung fibroblasts (Ohba et al., 1994). However, one of the ligands PDGF-C is mainly expressed and secreted by macrophages (Glim et al., 2013) and epithelial cells (Klinkhammer et al., 2018), and bleomycin increases the expression of PDGF-C mRNA in the lung (Zhuo et al., 2004). Taken together with our findings, there may be an interplay common to amiodarone and bleomycin that initial microinjuries to the alveolar epithelium by the toxic agents provoke inflammatory recruitment of macrophages. The dimer ligand PDGF-CC from macrophages and epithelia acts on fibroblasts to cause transformation to myofibroblasts via PDGFRα in parallel with the upregulation of PDGFRα by thrombin leaked from damaged blood vessels. Dabigatran shuts off the thrombin signal upregulating PDGFRα by inhibiting PARs.

In conclusion, amiodarone-induced ILD can be inhibited in the presence of a direct thrombin inhibitor. Investigation of the downstream signal indicated the involvement of fibroblast PDGFRα in the beneficial effect. Direct inhibition of PDGFRα might be a promising target for ILD in humans.

## Data Availability

The datasets presented in this study can be found in NCBI, accession number GSE162229.
