# Supplementary material for: Drug Repurposing Prediction and Validation From Clinical Big Data for the Effective Treatment of Interstitial Lung Disease
Source: Front Pharmacol. 2021 Sep 21;12:635293. doi: 10.3389/fphar.2021.635293 (PMC8490809; doi:10.3389/fphar.2021.635293)
Supplement: Supplementary file 3 [file DataSheet1.docx]

**Supplementary Material**


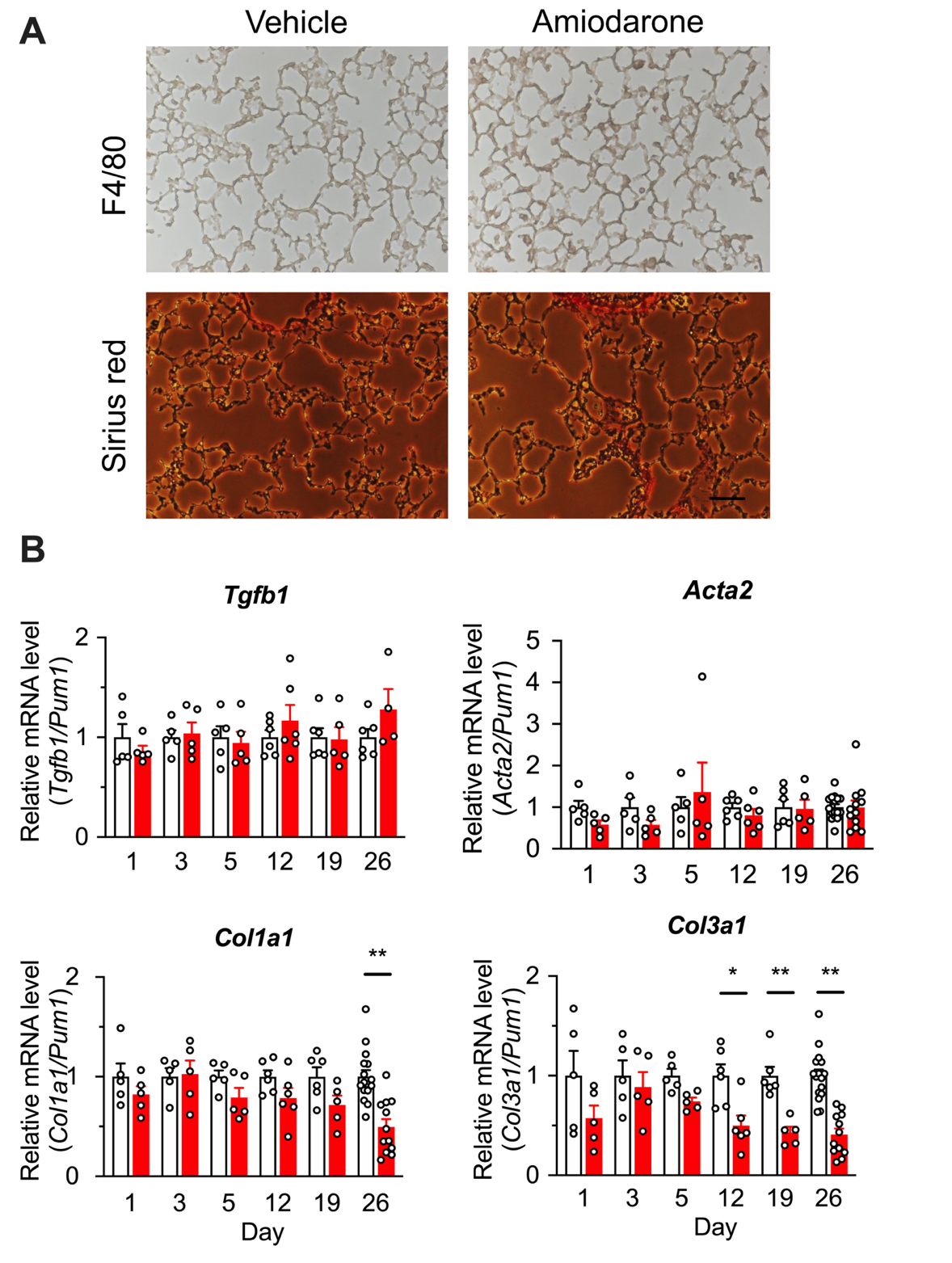


**Supplementary Fig. S1** **A** anti-F4/80 antibody and Sirius red staining of mouse lung section on day 26 after chronic oral treatment with amiodarone (300 mg⸱kg^–1^⸱day^–1^) or vehicle. Scale bar = 50 μm. **B** Quantitative RT-PCR results showing the time-course of expression of fibrosis-associated genes in the lung of mice after repetitive, daily treatment with amiodarone (red bar) or vehicle (white bar). *n* = 5, **P* < 0.05, ** *P* < 0.01 (two-way ANOVA with multiple comparisons).

Supplementary Table S1.xlsx

**Supplementary Table S1** Overall results of disproportionality analysis for interstitial lung disease (ILD) in the FDA Adverse Event Reporting System (FAERS) data

Individuals in the FAERS data were divided into the following four groups: (a) individuals who received the drug of interest (drug A) and exhibited ILD; (b) individuals who received the drug A, but did not exhibit ILD; (c) individuals who did not receive the drug A and exhibited ILD; and (d) individuals who did not receive the drug A and did not exhibit ILD. The reporting odds ratio (ROR) with 95% confidence interval (CI) and *Z* score was calculated as per formulae 1–3:

$ROR = \frac{\frac{a}{b}}{\frac{c}{d}}$…………………………………………………..…. 1

$95\% \mathrm{CI} = \exp\left\{ \log\left( ROR \right)\pm1.96\sqrt{\frac{1}{a}+\frac{1}{b}+\frac{1}{c}+\frac{1}{d}} \right\}$………….. 2

$Z \mathrm{score} = \frac{log(ROR)}{\sqrt{\frac{1}{a}+\frac{1}{b}+\frac{1}{c}+\frac{1}{d}}}$…………..…………..…………..……….. 3

where *a*, *b*, *c*, and *d* refer to the number of individuals in each group, and log refers to the common logarithm.

Supplementary Table S2.xlsx

**Supplementary Table S2** Overall confounding effects of concomitant drug (drug B) on amiodarone-induced interstitial lung disease (ILD) in the FDA Adverse Event Reporting System (FAERS) data.

Individuals who received amiodarone were divided into the following four groups: (a1) individuals who received the concomitant drug of interest (drug B) and exhibited ILD; (b1) individuals who received drug B, but did not exhibit ILD; (c1) individuals who did not receive drug B and exhibited ILD; and (d1) individuals who did not receive drug B and did not exhibit ILD. The reporting odds ratio (ROR) with 95% confidence interval (CI) and *Z* score for amiodarone-induced ILD was calculated as per formulae 4–6:

$ROR = \frac{\frac{a1}{b1}}{\frac{c1}{d1}}$…………………………………………………..…. 4

$95\% \mathrm{CI} = \exp\left\{ \log\left( ROR \right)\pm1.96\sqrt{\frac{1}{a1}+\frac{1}{b1}+\frac{1}{c1}+\frac{1}{d1}} \right\}$………….. 5

$Z \mathrm{score} = \frac{log(ROR)}{\sqrt{\frac{1}{a1}+\frac{1}{b1}+\frac{1}{c1}+\frac{1}{d1}}}$…………..…………..…………..……….. 6

where *a1*, *b1*, *c1*, and *d1* refer to the number of individuals in each group, and log refers to the common logarithm. Effect of drug B alone on the ROR of ILD was also analyzed as indicated in Supplementary Table 1 with the number of patients in 4 divided groups from *a0* to *d0*.

**Supplementary Table S3** Oligodeoxynucleotide primer sequences for RT-PCR

*Pum1* 5′-GCT TGT GCA GGA TCA GTA TGG-3′

5′-CAG CAC GCT CTG TAC GTG A -3′

*Pdgfra* 5′-TCA ACC CTA GTT CCT GCA TCC-3′

5′-GCT TGC AGA TCA TCC AGT CG-3′

*Pdgfc* 5′-AGT ACC ATG AGG TCC TTC AGT TGA G-3′

5′-TCC TGC GTT TCC TCT ACA CAC-3′

*Mmp12* 5′-GGT GGT ACA CTA GCC CAT GC-3′

5′-AGG AGG AGG TTT GTG CCT TGA-3′

*Timp1* 5′-TCA TCA CGG GCC GCC TAA-3′

5′-GTA CGC CAG GGA ACC AAG AA-3′

*Tgfb1* 5′-GCA ACA ATT CCT GGC GTT ACC-3′

5′-TAT TCC GTC TCC TTG GTT CAG C-3′

*Acta2* 5′-CTT TCA TTG GGA TGG AGT CAG C-3′

5′-CAA TGC CTG GGT ACA TGG TG-3′

*Cola1* 5′-AGA GGC GAA GGC AAC AGT CG-3′

5′-GCA GGG CCA ATG TCT AGT CC-3′

*Col3a1* 5′- ACC AAA AGG TGA TGC TGG AC-3′

5′-GAC CTC GTG CTC CAG TTA GC-3′
